# Deciphering the virulence factors, regulation, and immune response to *Acinetobacter baumannii* infection

**DOI:** 10.3389/fcimb.2023.1053968

**Published:** 2023-02-23

**Authors:** Afreen Shadan, Avik Pathak, Ying Ma, Ranjana Pathania, Rajnish Prakash Singh

**Affiliations:** ^1^ Department of Microbiology, Dr. Shyama Prasad Mukherjee University, Ranchi, Jharkhand, India; ^2^ Department of Biosciences and Bioengineering, Indian Institute of Technology, Roorkee, India; ^3^ College of Resources and Environment, Southwest University, Chongqing, China; ^4^ Department of Bioengineering and Biotechnology, Birla Institute of Technology, Ranchi, Jharkhand, India

**Keywords:** immune response, outer membrane protein, pathogenicity, virulence, antibiotics

## Abstract

Deciphering the virulence factors, regulation, and immune response to *Acinetobacter baumannii* infection*Acinetobacter baumannii* is a gram-negative multidrug-resistant nosocomial pathogen and a major cause of hospital acquired infetions. Carbapenem resistant *A. baumannii* has been categorised as a Priority1 critial pathogen by the World Health Organisation. *A. baumannii* is responsible for infections in hospital settings, clinical sectors, ventilator-associated pneumonia, and bloodstream infections with a mortality rates up to 35%. With the development of advanced genome sequencing, molecular mechanisms of manipulating bacterial genomes, and animal infection studies, it has become more convenient to identify the factors that play a major role in *A. baumannii* infection and its persistence. In the present review, we have explored the mechanism of infection, virulence factors, and various other factors associated with the pathogenesis of this organism. Additionally, the role of the innate and adaptive immune response, and the current progress in the development of innovative strategies to combat this multidrug-resistant pathogen is also discussed.

## Introduction

*A. baumannii* is a gram-negative, obligate aerobe, coccobacillus, and most commonly linked to global hospital-acquired infections (HAI) (Lin and Lan, 2014). Many of the infections related to the skin, soft tissues, bloodstream, and urinary tract are associated with this pathogenic organism (Peleg et al., 2008). A. *baumannii* has emerged as a multidrug-resistant (MDR) pathogen worldwide (Antunes et al., 2014) , and becomes prevalent across many civilian hospitals *via* cross-infection of already infected people and soldiers (Peleg et al., 2008). Experimental shreds of evidence have shown that *A. baumannii* is resistant to β-lactams, fluoroquinolones, aminoglycosides, and colistin. The resistance mechanism is assisted by the presence of efflux pumps, β-lactamases, aminoglycoside-modifying enzymes, and target modifications (Potron et al., 2015; Chakravarty, 2020).

*Acinetobacter* species are widely found in various environments, including soil, wastewater, skin of humans, and animals (Maravić et al, 2016). In healthy individuals, they occupy the nose, ear, throat, conjunctiva, vagina, hand, groin, and toe webs (Al Atrouni et al., 2016). In hospitals, they reside on medical devices, syringes, medical equipment, tap water sinks, door handles, and dispensers (Dexter et al., 2015). Clinical infection with *Acinetobacter* is often seen in patients with underlying debilitating conditions and infections are often device-associated, including ventilator-associated pneumonia, and catheter-associated urinary tract infections (Jiang et al., 2021). *A. baumannii* is the major causative agent of ventilator-associated pneumonia, accounting for nearly 15% of all hospital-acquired infections (Demirdal et al., 2016), and it confers 26% mortality rate that goes up to 43% in ICU (Greene et al., 2016).

Most *A. baumannii* infections occur in the intensive care units (ICU) and account for approximately 20% of infections in ICUs globally (Vázquez-López et al, 2020; Kyriakidis et al., 2021). The ICU mortality of ventilator-associated pneumonia caused by MDR *A. baumannii* has been reported to be as high as 84.3% (Wisplinghoff et al., 2012). The emergence of antibiotic resistance is directly attributed to the magnitude of antibiotic consumption (Roy et al., 2022) and its resistance can be transmitted from one bacterium to another by means of horizontal gene transfer, which results in the transfer of resistance among different species (Read and Woods, 2014). It is assumed that the spread of infection will lead to approximately 300 million deaths by 2050 (Chokshi. et al, 2019). Although AMR in microorganisms is ancient, the evolution of resistance in human pathogens is a modern phenomenon driven by the frequent use of antibiotics (Davies and Davies, 2010; Larsen et al., 2022). Additionally, the survivability of the organism in adverse conditions like desiccation and extreme pH, makes it difficult in managing infection, especially in the intensive care and burns units of hospitals (Dexter et al., 2015). Additionally, the community-acquired infections (CAI) of *A. baumannii* have been increasing gradually. These CAI are most frequently found in hot and humid climate countries, and typically occur in individuals suffering from diabetes mellitus, pulmonary infections, heavy smokers, or heavy alcoholic drinkers (Falagas et al., 2007; Dexter et al., 2015).

Previous research studies employing genomics and phenotypic analyses have identified many virulence factors such as outer membrane proteins (OMPs), protein secretion systems, lipopolysaccharides (LPS), phospholipases, proteases, and iron (Fe)-chelating systems (McConnell et al., 2013; Lin and Lan, 2014). A previous study demonstrated the potential of *A. baumannii* to rapidly develop resistance to various antimicrobials and it leads to the development of multidrug-resistant strains (McConnell et al., 2013). The majority of *A. baumannii* infections are caused by healthcare tools or by contact with a person who has been exposed to the bacterium through contact with another infected patient (Asensio et al., 2008; Rodriguez-Bano et al., 2008). Experimental evidence has shown that *A. baumannii* is implicated in infections of the bloodstream in 10 to 15% of cases, however, the origin of the infection is unclear (Garnacho-Montero et al., 2015). *A. baumannii* is a major threat to neurosurgery patients and is related to 4% of meningitis, and shunt-related infections with a more than 70% mortality rate (Basri et al., 2015).

Due to the similarity among closely related species, distinguishing the *Acinetobacter* taxonomy only using phenotypic properties is not easy due to the very high similarity among closely related species, however, developments in many fingerprinting methods like matrix-assisted laser desorption ionization-time of flight (MALDI-TOF), pulse-field gel electrophoresis (PFGE), repetitive extragenic palindromic sequence-based polymerase chain reaction (rep-PCR), multilocus sequence typing (MLST), and RNA spacer fingerprinting made it convenient to tying among the closely related strains (Chang et al, 2005; Lee et al., 2015; Li et al., 2015).

## Virulence and pathogenesis factors

Pathogenesis is a qualitative term that denotes the ability of a microorganism to establish infection in a host. On the other hand, virulence is a quantitative term that represents the degree of host damage caused by a pathogen. However, the factors that are responsible for the establishment of the infection, *i.e.* pathogenesis, also determine the extent of the infection *i.e.* virulence. *A. baumannii* has got a specialized arsenal of virulence factors that provide fitness advantages at different stages of pathogenesis, starting from survival under the host immune response to attachment, internalization, and apoptosis of host cells (Harding et al., 2017b). Outer membrane proteins (OMPs) such as OmpA help in attachment and internalization into host epithelial cells. In addition to this, OmpA induces secretion of apoptotic factors inside the host cell that initiates the apoptosis process leading to cell death (Choi et al., 2008a; Choi et al., 2008; Moon et al., 2012; Zhang et al., 2022). Capsular exopolysaccharides in *A. baumannii* protect the pathogen from environmental and host-mediated stresses, and the composition determines the degree of virulence (Tipton and Rather, 2017; Singh et al., 2019; Talyansky et al., 2021). Similarly, the pathogen has specialised metal ion uptake system to counteract the host mediated metal ion chelation called nutritional immunity (Archibald and Fridovich, 1981; Green et al., 2020). In addition to these, multiple classes of efflux pumps are present which also participate in motility and attachment, apart from their bonafide work of multidrug efflux. Major virulence factors of *A. baumannii* and their role in pathogenesis are summarized in **Table 1**.

**Table 1:** Major virulence factors of *A. baumannii* along with their role in pathogenesis.

Virulence factors	Role in pathogenesis	Reference
Autotransporter (Ata)	Support adherence and biofilm development	Thibau et al. (2019)
AbeD	Host cells killing	Srinivasan et al. (2015)
AdeRS	Regulator of virulence	Montaña et al. (2015)
BaeSR	Virulenceregulator	Lin et al. (2014)
BfmRS	Virulenceregulator/Csu pili expression	Kim et al. (2009)
Biofilm associate proteins	Adherence and biofiolm development	Brossard and Campagnari (2011)
BAP like proteins (BAP)	Enhancement of adherence	De Gregorio et al. (2015)
Capsular polysaccharides	Enhance bacterial survivality in tissues and biofilm formation	
CipA	Enhance serum resistance and promote tissue invasion	Koenigs et al. (2016)
CheAY	Virulenceregulator/Csu pili expression	Chen et al. (2017)
FhaBC	Promote the adherence in tissue and host cells killing	Pérez et al (2017)
GacS	Promote neutrophil influx	Bhuiyan et al. (2016)
GigABCD	Support *in vivo* survivality and host cells killing	Gebhardt et al. (2015)
Iron acquisition system	Support *in vivo* survivality and host cells killing	Megeed et al. (2016)
Lipopolysaccharides (LPS)	Evasion of host immune system and tissue infection	Lees-Miller et al. (2013)
MumC/MumT	Support *in vivo* survival	Juttukonda et al. (2016)
OMVs	carry virulence factors and antibiotic resistance gene	Li et al. (2016)
PER-1	Support *in vivo* survival and serum resistance	Russo et al. (2009)
Penicillin binding protein7/8 and PER-1	Support adherence and *in vivo* survival	Lee et al. (2008)
Pilli	Promote adherence and biofilm formation	Tomaras et al. (2008)
PLC/PLD	Support *in vivo* survival and serum resistance	Fiester et al. (2016)
PmrAB	Antimicrobial resistance and LPS modification	Beceiro et al. (2011)
Porins (OmpA/OMP 33-36,Omp22)	Promote tissue adherence and invasion	Huang et al. (2016)
RecA	Support *in vivo* survival	Aranda et al. (2011)
SurA1	Support *in vivo* survival and serum resistance	Liu et al. (2016)
Type I secretion system	Enhance biofilm formation	Harding et al. (2017b)
Type II secretion system	Support *in vivo* survival	Harding et al. (2016)
Type V secretion system	Promote adherence and biofilm formation	Bentancor et al. (2012b)
Type VI secretion system	Killing of competitor bacteria and	Ruiz et al. (2015)
	support host colonization	
Tuf	Enhance serum resistance	Koenigs et al. (2015)
UspA	Support *in vivo* survivality and host cells killing	Gebhardt et al. (2015)
ZnuABC, ZigA, ZrlA	Enhance *in vivo* survival and persistence	Lonergan et al. (2019)
(Zinc acquisition system)		

## OMPs

**Figure 1:**
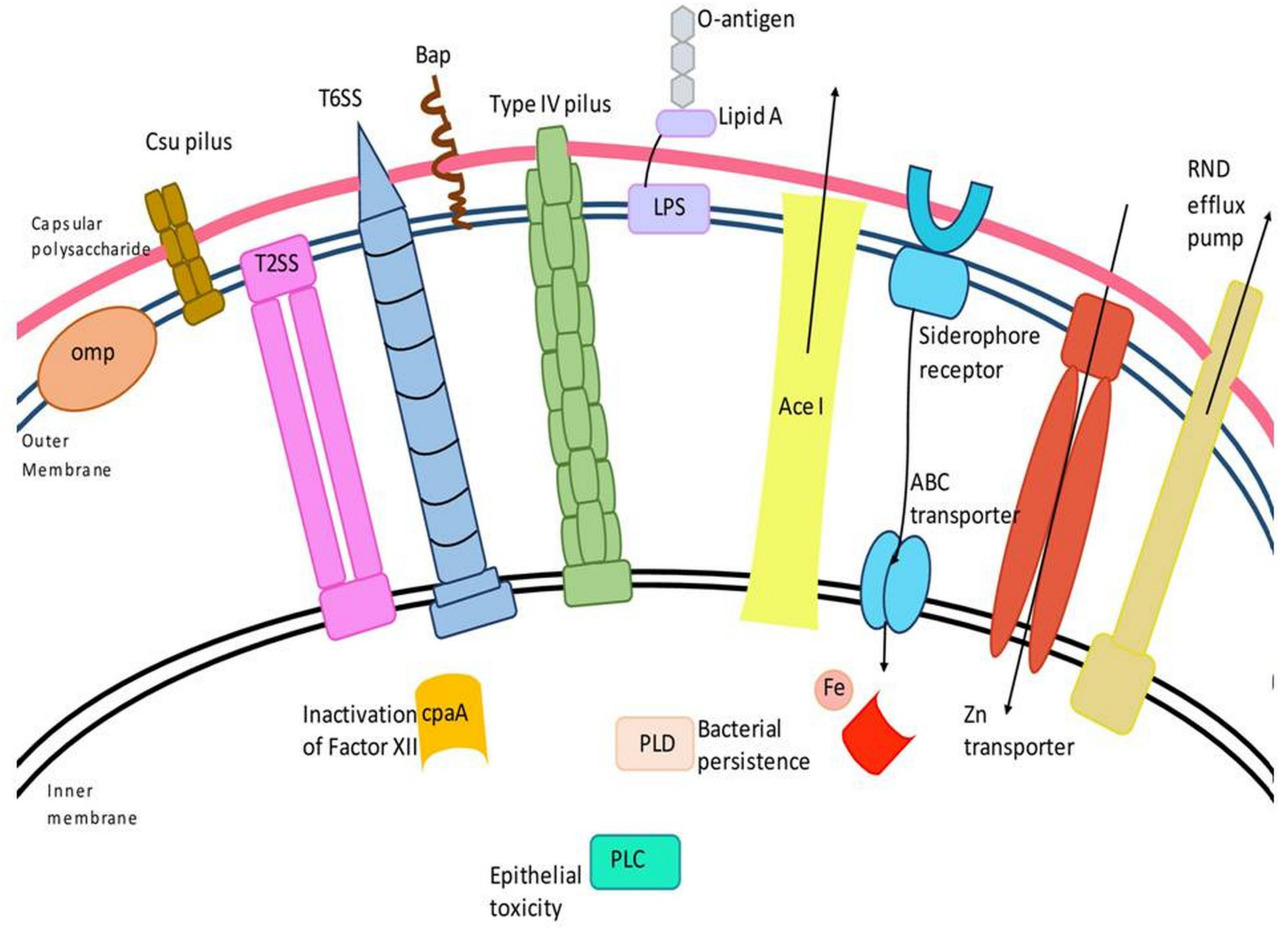
Schematic representation of major virulence factors of *A. baumannii*. *A. baumannii* has several virulence factors that enable the bacterium to confer pathogenic resposne, adhere, and invade host cells. Virulence factors include the outer membrane protein, Type II and VI secretion systems, phospholipase D (PLD), capsular polysaccharide, Csu (chaperon/usher) pilus system, AceI (*Acinetobacter* chlorhexidine) efflux protein, the inner membrane two-component system, efflux-pumps and lipopolysaccharide, etc.

OMPs are localized in the outer membrane of gram-negative bacteria and collectively form a critical factor in virulence (**Figure 1**). In *A. baumannii*, they play a diverse role starting from attachment to the host cell surface to the induction of apoptosis in the host cell *via* activation of caspases (Choi et al., 2005). In addition, they also mediate resistance to multiple classes of antibiotics and host-mediated stresses (Smani et al., 2014). Collectively, they aid in the survival of *A. baumannii* in the host and its success as a multi-drug resistant nosocomial pathogen. The major OMPs that play a key role in the pathogenesis and virulence of *A. baumannii* are OmpA, CarO, outer membrane carboxylate channels (Occ), OmpW, and Omp33-36.

OmpA is the most abundant porin in the outer membrane of *A. baumannii* that consists of two different structural domains *i.e.* an N-terminal, eight-stranded antiparallel β-barrel in the outer membrane and a globular C-terminal periplasmic domain that interacts with the cell wall (Moon et al., 2012; Iyer et al., 2018). OmpA is involved in eukaryotic cell adhesion, invasion, apoptosis and antibiotic resistance, and is a promising candidate for vaccine development (Choi et al., 2005; Smani et al., 2014; Ansari et al., 2018). OmpA interacts with Toll-like receptor 2 (TLR2) in lung epithelial cells. It causes downregulation and internalization of E-cadherin, an important adhesion junction protein, thereby increasing lung epithelial permeability. It also causes actin reorganization in lung epithelial cells (Zhang et al., 2022).

Once inside the cytoplasm, OmpA gets fragmented and due to the presence of nuclear localization signal (KTKEGRAMNRR) some fragments are transported to the nucleus, which causes fragmentation of the nuclear DNA. DNaseI like enzymatic activity was observed with OmpA, as the purified OmpA caused chromosomal degradation when injected into fertilized *Xenopus laevis* eggs. In addition, when OmpA was incubated with supercoiled plasmid DNA, the proportion of linear and relaxed forms of plasmid increased in a time-dependent manner. The DNaseI activity of OmpA is also dependent on the presence of Ca^2+^ and Mg^2+^ (Choi et al., 2008).

OmpA also gets localized into the mitochondria and induces apoptosis *via* a complex mechanism that involves destabilization of mitochondrial membrane potential followed by the release of proapoptotic compounds such as cytochrome c and Apoptosis-inducing factor (AIF) into the cytosol, which activates the caspases (Choi et al., 2005). Outer membrane vesicles (OMVs) secreted by *A. baumannii* also carry a significant proportion of OmpA. These vesicles fuse with the host cell membrane at cholesterol-rich regions and release the cargo inside the cell, including OmpA. The N-terminal region of OmpA is necessary for the induction of apoptosis and OMVs derived from a *A. baumannii ompA* deletion mutant failed to induce apoptosis in macrophages (Jin et al., 2011). In addition, OmpA also plays an important role in biofilm formation and resistance to antibiotics such as chloramphenicol, aztreonam, and nalidixic acid (Gaddy et al., 2009; Smani et al., 2014).

CarO, or carbapenem susceptibility protein, was first identified in an *A. baumannii* strain, where the deletion of the gene encoding a 29 kDa protein conferred resistance to imipenem (Limansky et al., 2002). Several reports thereafter have indicated its role in carbapenem resistance as well as the uptake of amino acids such as ornithine and glycine (Mussi et al., 2007; Labrador-Herrera et al., 2020; Uppalapati et al., 2020). In addition to its possible role in carbapenem resistance, *CarO* also mediates adhesion and invasion into epithelial cell lines. The deletion mutant of *carO* is compromised in adherence and invasion into a lung epithelial cell line as compared to wild type (WT) *A. baumannii* (ATCC 17978). In addition, the bacterial burden in organs of mice infected with the *carO* mutant was significantly less than in mice infected with WT *A. baumannii* ATCC (17978). Complementation with a functional copy of carO restored the adhesion, invasion, and the *in vivo* dissemination ability (Labrador-Herrera et al., 2020)

Outer membrane carboxylate channels (Occ) form monomeric 18 stranded beta barrels and are involved in the transport of small molecules owing to the narrow pore size of the channels. There are five orthologs of Occ proteins in *A. baumannii* ATCC (17978), and are namedOccAB1 (formerly OprD), OccAB2, OccAB3, OccAB4, and OccAB5 (Zahn et al., 2016). OccB1 shares 49% similarity to *Pseudomonas aeruginosa* OprD which is also a member of Occ family proteins in *P. aeruginosa* (Dupont et al., 2005). OprD is involved in the uptake of carbapenems in *P. aerugenosa* and the deletion of *oprD* results in a significant decrease in the susceptibility to imipenem, and meropenem (Sakyo et al., 2006). However, in *A. baumannii*, the OccAB1 is not involved in carbapenem uptake as deletion of occAB1 did not show any significant change in MIC of imipenem, meropenem, colistin, ceftazidime and ciprofloxacin, when compared to the WT strain (Catel-Ferreira et al., 2012). OccAB1 might be involved in the uptake of Fe^2+^ and Mg^2+^ during adaptation to iron and/or magnesium depleting condition, a function that is similar to *P. aerugenosa* OprQ (Catel-Ferreira et al., 2012). A novel OprD homolog in *A. baumannii* was found to be associated with hypervirulence in CRAB (carbapenem-resistant *A. baumannii*) clinical isolates (Hua et al., 2021).

OmpW forms an eight-stranded β barrel in the outer membrane and plays an important role in adhesion to pulmonary epithelial cell lines and subsequent invasion. Deletion of *ompW* significantly compromised the ability of *A. baumaannii* to adhere and invade the alveolar basal epithelial A549 cell line. The deletion mutant of *ompW* is also compromised in its ability to form biofilms (Catel-Ferreira et al., 2016; Gil-Marqués et al., 2022). Down-regulation of *ompW* in colistin-resistant cells led to the hypothesis of the involvement of the encoding protein (OmpW) in conferring resistance to colistin. However, a study found that the MIC of colistin was the same for the deletion mutant of *ompW* and the *A. baumannii* ATCC 17978 WT strain which rules out the possibility of colistin resistance mechanism, depending uniquely on ompW downregulation. Increased expression of *ompW* during iron abundance indicates its role in iron uptake (Nwugo et al., 2011; Catel-Ferreira et al., 2016). The expression of *ompW* is transcriptionally regulated by BfmRS two component system, which also regulates the expression of multiple stress response genes including genes involved in oxidative stress response, osmotic stress response, and siderophore production (Geisinger et al., 2018).

Omp33-36 of *A. baumannii* is associated with carbapenem resistance, adhesion and invasion into epithelial cells, and cytotoxicity. The deletion mutant of *omp33* displays lower adhesion and invasion in the A549 cell lines as compared to ATCC 17978 cells. Once inside the cell, Omp33-36 activates caspases, which leads to host cell apoptosis. In contrast to OmpA, which also induces apoptosis in host cells, Omp33-36 lacks the nuclear localizatin signal (Rumbo et al., 2014). Omp33-36 is important for the induction of cell death in epithelial and macrophage cells. A549 and macrophage cells infected with *omp33* deletion mutant, they showed greater survival compared to cells infected with the WT *A. baumannii* ATCC 17978. In a mice infection model, the LD50 of the *ompA* deletion mutant was also found to be higher when compared to the WT strain (Smani et al., 2013).

## Pili

Pili are short antigenic, hair-like appendages that are found on the surface of both gram-positive and gram-negative bacteria. They play a very important role in the attachment to biotic and abiotic surfaces, biofilm formation, motility and conjugation in a class-dependent manner. In gram-negative bacteria, there are five classes of pili: (i) chaperone-usher pili (ii) type IV pili (iii) conjugative type IV secretion pili (iv) curli fibers, and (v) type V pili (Hospenthal et al., 2017; Ramezanalizadeh et al., 2020). In *A. baumannii*, four classes of chaperone-usher type I pili have been identified including the *csu* gene cluster, which has been studied extensively (Eijkelkamp et al., 2014). The *csu* gene cluster encodes four different proteins namely CsuA/B, CsuA, CsuB, and CsuE, which are assembled with the help of CsuC and CsuD, representing the chaperone and the usher, respectively. CsuA/B forms the shaft and CsuE forms the tip of the appendage (Tomaras et al., 2003; Pakharukova et al., 2015). CsuE mediates attachment to hydrophobic substrates owing to the presence of three hydrophobic finger loops at its N-terminal domain. Mutations in the finger loops that decrease hydrophobicity resulted in a significant decrease in biofilm formation (Pakharukova et al., 2017). Csu pili facilitate tight attachment to abiotic, hydrophobic surfaces, and very loose attachment to hydrophilic surfaces (**Figure 1**) (Pérez-Varela et al., 2019). Csu pili, however, do not enable bacteria to attach to epithelial cell lines (de Breij et al., 2009).

The origin of the term *Acinetobacter* is a Greek word i.e. “*akineto*,” which means non-motile. However, many *A. baumannii* strains show motility through two different mechanisms; twitching and surface-associated motility. The ability to move by twitching or surface-associated motility depends on the individual strain. Twitching motility is a form of appendages-dependant and not dependant movement that is driven by sequential extension, attachment, and retraction of type IV pilli (Aranda et al, 2014; Chlebek et al., 2019). It is associated with surface attachment and biofilm formation employing *pilA, pilD*, and *pilT* genes (Dhabaan et al., 2016), and the GacS/GacA two-component regulatory system (Cerqueira et al., 2014). In addition to their role in twitching motility, type IV pili also mediate natural transformation (Harding et al, 2013). The major pilin subunit, PilA is encoded by the *pilA* gene. Variability in the *pilA* gene among *A. baumannii* isolates leads to differences in surface chemistry, for example, the surface electrostatic charge of the pilin subunits which maintains a balance between motility and biofilm formation. For example, *pilA* of *A. baumannii* ACICU promotes biofilm formation, whereas *pilA* of *A. baumannii* AB5075 promotes motility (Ronish et al., 2019).

Curli are amyloid fibers and a major component of the extracellular matrix that play an important role in biofilm formation. CsgA is the major structural component and is transported to the cell surface by CsgG (Hospenthal et al., 2017). In *E. coli*, there are two operons (*csgBAC* and *csgDEFG*), that encode proteins required for the synthesis of curli fibers. In *A. baumannii*, the gene encoding an ortholog of CsgG is negatively regulated by a plasmid pAB5, and disruption of *csgG* results in a significant decrease in the thickness of extracellular matrix, and also affects biofilm formation (Di Venanzio et al., 2019; Benomar et al., 2021). However, in *Acinetobacter* sp. a complete curli biosynthetic machinery is absent and curli fibers have not been reported yet (Benomar et al., 2021).

## LPS

LPS is a major constituent of the outer membrane of most gram-negative bacteria. It provides structural integrity and acts as a permeability barrier for hydrophobic small molecules (Nikaido and Vaara, 1985; Carpenter et al., 2016). In addition to these functions, due to its high abundance in the outer membrane, LPS is recognized as a microbe associated molecular pattern (MAMP) by host immune cells (Needham and Trent, 2013; Bertani & Ruiz, 2018). Structurally, LPS consists of three components: lipid A, an oligosaccharide core as well as a repetitive O-antigen. Lipid A is a glycolipid that consists of two B 1-6 linked glucosamine and lipid tails that are anchored into the outer leaflet of the outer membrane. The core oligosaccharide is a non repeating carbohydrate chain that connects lipid and the O antigen, a major immunogen consisting of repeating oligosaccharides. LPS that comprises all three regions is known as smooth LPS, whereas lipooligosaccharide or (LOS) that lacks the O antigen is referred to as rough LPS (Bertani and Ruiz, 2018). The core oligosaccharide is hydrophilic and acts as a barrier to hydrophobic moieties. Lipid A tends to carry 4 -7 saturated fatty acid chains that aid in the membrane stability and hydrophobic barrier properties. However, the negatively charged phosphate groups tend to repel each other which are further stabilized by divalent cations such as Mg^2+^ (Nikaido and Vaara, 1985; Bertani and Ruiz, 2018).

*A. baumannii*, LPS induces the expression of several pro-inflammatory cytokines in the host. These includes TNFα, IL8, CCL4, and the LOS receptor TLR4 (Kikuchi-ueda et al, 2021). Mutation in *lpxB*, involved in LPS biosynthesis resulted in a significant reduction in surface-associated motility in *A. baumannii* 307-0294. Motility was restored when the mutant was complemented with a functional copy of *lpxB*. This indicates the involvement of LPS in surface-associated motility (McQueary et al., 2012). LPS shedding in the course of infection also determines the magnitude of immune cell activation. In a study, it was found that culture supernatant from highly virulent clinical strains was more potent in the activation of TLR4 signaling compared to *A. baumannii* ATCC 17978. However, in its purified form, LPS from ATCC 17978 was more potent in activating TLR4 compared to the virulent clinical strains (Lin et al., 2012).

Similarly, inhibition of the enzyme involved in lipid-A biosynthesis ‘LpxC’ suppresses the LPS-mediated TLR4 activation in *A. baumannii.* The LpxC inhibition enhanced the clearance of *A. baumannii* through phagocytic killing and was found to affect virulence. *A. baumannii* cells treated with LpxC inhibitor showed lower bacterial burden in tissues compared to the untreated control (Lin et al., 2012). The LPS is responsible for the susceptibility of *A. baumannii* to colistin, a cationic polypeptide that interacts with the negatively charged lipid-A. Mutations in either of the genes *lpxA, lpxC*, and *lpxD* involved in lipid-A biosynthesis resulted in a complete loss of LPS. The strains that are deficient in LPS show less membrane integrity compared to the wild-type strains. They however become resistant to colistin (Moffatt et al., 2010).

## Capsular exopolysaccharide or capsule

The capsule, or capsule, is a major virulence factor that comprises tightly packed repeating oligosaccharides subunits (K Units) and forms a protective layer on the bacterial surface. CPS assists the bacteria in the evasion of the host immune system, enhances resistance to antimicrobial compounds and helps withstand prolonged desiccation. Resistance to different antimicrobial compounds and the ability to survive desiccation are major reasons behind the success of *A. baumannii* as a MDR nosocomial pathogen (Geisinger and Isberg, 2015; Tipton and Rather, 2017; Harding et al, 2017b). The abolition of the capsule from an otherwise capsule producing strain significantly affects its virulence (Russo et al., 2010).

The genes that encode the proteins required for the synthesis and transport of capsular polysaccharides are organised in capsule locus (KL) which is present between fkpA and lldP genes, and varies in length from 20 to 35 kb. Till date over 100 different types of capsule loci have been identified in different *A. baumannii* strains (Shashkov et al., 2017).The diversity of capsular locus translates into the diversity of the K units which vary in length as well as in composition. They contain derivatives of simple UDP-linked sugars such as glucose, galactose and/or complex sugars such as non-2-ulosonic acids. The length also varies from 2 (K53) to 6 (k37) monosaccharide units and individual K unit can be linear or branched (Arbatsky et al., 2015; Kenyon et al., 2016a; Kasimova et al., 2017; Shashkov et al., 2018). Exposure to certain antibiotics, such as chloramphenicol at a sub-MIC concentration has been found to increase the production of capsule with a concomitant increase in survival against complement mediated killing and increased virulence in mice infection model (Geisinger and Isberg, 2015). This increase in capsule production was due to an increase in the expression of genes from the K locus, and was found to be reversible and non-mutational. BfmRS two component system was found to be important for this upregulation, since there was no significant change in the expression of K locus genes in the *bfmRS* deletion mutant upon exposure to similar concentrations of chloramphenicol (Geisinger and Isberg, 2015).

The composition of the K units greatly influences the virulence. In a recent study, it was found that disruption of the gene *gtr6* that resulted in the inactivation was responsible for the hypervirulent trait of *A. baumannii* strain HUMC1. The functional copy of the gene in *A. baumannii* strain ATCC 17978 encodes for a glycosyltransferase that adds an N-acetyl-β-D-glucosamine to the K unit. Upon disruption of the gtr6 gene in ATCC 17978, the N-acetyl-β-D-glucosamine sugar was lost from the K unit and the mutant strain became hypervirulent. Disruption of the *gtr6* gene resulted in a decrease in phagocytosis by macrophages and also a marked increase in blood bacterial burden in a bacteremia mouse model, compared to the strain with a functional copy of *gtr6*. The mutation, however, resulted in no change in capsule abundance (Talyansky et al., 2021).

## Efflux pumps

The low permeability of the outer membrane of *A. baumannii* makes it difficult for large sized antibiotics to gain entry into the cell. In addition to this, there are different classes of efflux pumps that extrude the antibiotics. There are six classes of efflux pumps namely ATP binding cassette (ABC) transporter, major facilitator superfamily (MFS), resistance nodulation division (RND), multidrug and toxic compound extrusion (MATE), proteobacterial antimicrobial compound efflux (PACE) and small multidrug resistance (SMR) family. ABC transporters are dependent on the hydrolysis of ATP as an energy source, others use hydrogen ion gradient to transport antimicrobials out of the cell (Jordi Vila, Servei, 2007; Kornelsen, 2021). In *A. baumannii*, the resistance due to efflux can majorly be attributed to RND and MFS family transporters (Ogata et al., 2006).

The MFS transporter family is the most diverse and present in all domains of life. They consist of 12-14 transmembrane helices divided into two domains present in the inner membrane. These transporters can be divided into three broad categories: (i) symporters that transport two compounds in the same direction (ii) antiporters that transport two compounds in two different directions; and (iii) uniporters that transport only a single compound along its concentration gradient. For symporters and antiporters, at least one compound must move along its concentration gradient, which is used as an energy source to transport the other compound (Du et al., 2018; Kornelsen, 2021). Some important members of this family present in *A. baumannii* are chloramphenicol resistance *Acinetobacter*, or CraA, which confers resistance to chloramphenicol; AbaQ, which confers resistance to quinolones; and AbaF, which is involved in resistance to fosfomycin (Roca et al., 2009; Sharma et al., 2017; Kornelsen, 2021). In addition, there are Tet-efflux pumps such as TetA and TetB that mediate resistance to tetracycline and whose encoding genes are present within transposable elements or on plasmids, and are easily acquired *via* horizontal gene transfer (Ribera et al., 2003; Srinivasan et al., 2009). Intrinsic resistance to broad classes of antibiotics as observed in multiple gram-negative bacteria can be attributed to the presence of RND family efflux pumps which are involved in the efflux of nearly all classes of antibiotics (Coyne et al., 2011; Kornelsen, 2021). The majority of the members of this family form a tripartite complex involving a transporter, a membrane fusion protein, and an outer membrane protein (Coyne et al., 2011; Du et al., 2018; Kornelsen, 2021).

The first RND efflux pump (AdeABC) discovered in *A. baumannii* belongs to the most clinically relevant RND family pump (Coyne et al., 2011; Kornelsen, 2021). AdeABC confers resistance to multiple classes of antibiotics including aminoglycosides, chloramphenicol, beta-lactams, erythromycin, and fluoroquinolones etc. The transporter consists of three components: AdeA, a membrane fusion protein; AdeB, the transporter, and AdeC, an outer membrane porin. In absence of AdeC, the membrane fusion protein and the transporter can also interact with other OMPs such as AdeK. Expression of this transporter is regulated by AdeRS, a two component signaling system (Magnet et al., 2001; Jordi Vila, Servei, 2007; Kornelsen, 2021). Compared to other RND family efflux pumps, AdeABC is found to be overexpressed in the largest number of clinical MDR isolates making it the major contributor to MDR phenotype (Coyne et al., 2011; Yoon et al., 2013; Zhu et al., 2020). Other important members of this family are AdeFGH, AdeIJK, AbeD, and AcrAB amongst others (Du et al., 2018; Kornelsen, 2021). AbeD is an orphan efflux pump that mediates resistance to rifampicin, gentamicin, ceftriaxone, and tobramycin. In addition, it is also involved in mediating resistance to osmotic stress, oxidative stress, and pH changes, etc. Deletion of AbeD was found to affect survival under oxidative stress and osmotic stress, and also affected motility. The cognate membrane fusion protein and OMPs are yet to be discovered (Srinivasan et al., 2015).

Other than their established role as multidrug efflux proteins, members of RND, MATE, SMR, and ABC superfamily are also involved in surface-associated motility and virulence. Deletion mutants of a few members of RND, MATE, SMR and ABC superfamily were found to be compromised in surface-associated motility compared to the WT strain. The mutants also showed reduced virulence in *C. elegans* and *G. mellonella* infection models (Pérez-Varela et al., 2019). Another study indicates that the efflux pump AdeFGH co-transport antibiotic and acyl homoserine lactone, an important autoinducer compound in *A. baumannii*. The efflux pump AdeFGH, thereby plays an important role in quorum sensing and the pathways that are regulated by quorum sensing, including biofilm formation (He et al., 2015).

## Metal ion acquisition systems

Iron is an essential metal ion for the survival of bacteria and the host implies every possible mechanism to restrict the availability of iron to bacterial pathogens. Most of iron is bound with the hemoglobin and the host recruits other proteins such as transferrin, lactoferin, or ferritin, to sequester the remaining free iron. An immune system protein (calprotectin) sequesters a plethora of metal ions including iron in ferrous form. The pathogen *A. baumannii* has multiple iron uptake mechanisms to meet its iron requirement that include a heme uptake system and siderophore production (Nakashige et al., 2015; Cook-Libin et al., 2022).

*A. baumannii* secretes phospholipase C (encoded by *plc1* and *plc2*) that causes the lysis of RBCs and the release of hemoglobin (Antunes et al., 2011; Ticak et al., 2016). The free hemoglobin is then taken up inside the cell by an active heme uptake system that consists of a secreted hemophore (HphA), a TonB-dependent outer membrane receptor (HphR), a periplasmic iron binding protein, an inner membrane ABC transporter, a putative heme oxygenase (HemO), and a few other components. The heme oxygenase converts Fe^3+^ to Fe^2+^ which can be used in cellular processes (Giardina et al., 2019; Bateman et al., 2021).

In addition to this, *A. baumannii* also employs three different classes of siderophores: acinetobactin, baumannoferrin, and fimsbactin. These three classes are functionally redundant to some extent as deletion of the genes responsible for the synthesis of any of them does not drastically affect the *in-vitro* iron chelation ability of the mutants (Sheldon and Skaar, 2020). Some strains, for example, *A. baumannii* ATCC 19606 produce only one class of siderophores (acinetobactin), which is sufficient to allow growth under iron-limiting conditions (Dorsey et al., 2004; Penwell et al., 2012; Cook-Libin et al., 2022). Their role in pathogenesis however is not similar. In the type strain *A. baumannii* ATCC 17978, acinetobactin has been found to be most important for growth in presence of human serum or human transferrin as the sole iron source. Similar to this, acinetobactin has been found to be more important than the other two in mice infection models (Sheldon and Skaar, 2020).

The energy needed for the siderophores-mediated iron uptake through the outer membrane is provided by the proton motive force across the inner membrane, and is mediated by TonB-ExbB-ExbD protein complex. Transport of the complex through the inner membrane mostly happens through specialised ABC transporters (Noinaj et al., 2010; Cook-Libin et al., 2022). In response to bacterial siderophores, the neutrophils secrete siderocalin that sequesters ferric catecholate and carboxymycobactin-type siderophores and prevents their uptake by the bacteria. To counteract the effect of siderocalin, bacteria secrete modified siderophores. However, the interaction of siderocalins with siderophores secreted by *A. baumannii* is yet to be investigated (Sheldon and Skaar, 2020).

Mn^2+^ is required in multiple physiological processes starting from DNA replication to protection against oxidative stress. Mn^2+^ acts as a co-factor of several enzymes such as superoxide dismutase that are involved in protection against reactive oxygen species (ROS) (Green et al., 2020). In addition, low molecular weight Mn-metabolite complexes can detoxify ROS in an enzyme-independent manner (Archibald and Fridovich, 1981; Barnese et al., 2012; Green et al., 2020). In response to host-calprotectin-mediated nutritional immunity, *A. baumannii* uses a high affinity resistance-associated macrophage protein (NRAMP) family Mn-transporter i.e. MumT (manganese and urea metabolism transporter). The gene encoding this transporter is a part of an operon that encodes genes involved in urea metabolism and is regulated by a LysR family transcriptional regulator, mumR (Juttukonda et al., 2016).

Deletion of *mumT* (Δ*mumT*) resulted in a significant loss in virulence as it was evident from a reduction in bacterial burden in the lungs and liver, in a mice pneumonia model. Interestingly, in a co-infection model, this defect was rescued in the liver but not in the lungs in calprotectin-deficient mice. The higher abundance of manganese in the liver compared to the lungs and the decreased fitness of Δ*mumT* in wild type mice indicates the importance of the high affinity Mn-transporter in the survival of *A. baumannii* during calprotectin-mediated nutritional immunity (Juttukonda et al., 2016). Deletion of the gene encoding the manganese-responsive transcriptional regulator MumR resulted in an increased sensitivity to H_2_O_2_-mediated killing, which was partially rescued upon supplementation with manganese (Green et al., 2020).

## Secretion systems

In *A. baumannii* various protein secretion systems have been discovered. The type I secretion system (T1SS) of *A. baumannii* is a homolog of the *E. coli* hemolysin exporter, TolC-HlyD-HlyB system. TISS is involved in the transport of Bap proteins and RTX-like serralysin toxin. Secretion through the Type II secretion system (T2SS) is a two step process, with the transport of effector proteins to the periplam by the secretory pathway (Sec) or the Twin-arginine (Tat) system followed by their secretion to the extracellular space. The 12 to 15 proteins of T2SS are assembled in 4 categories: a pseudopilus, a cytoplasmic ATPase, an inner-membrane complex assembly, and the outer complex made up of dodecameric proteins (Korotkov et al., 2012). The T2SS was first identified in the *A. baumanni* ATCC 17978 with the general secretory pathway distributed through the discrete clusters and not on a single operon (Eijkelkamp et al., 2014). Effector proteins including various enzymes such as alkaline phosphatase, elastase, lipase, and phospholipases play a key role in *A. baumanni* virulence (Elhosseiny and Attia, 2018).

In *A. baumanni*, specific T2SS lipase effectors such as LipA and LipH, hydrolyze long-chain fatty acids as the carbon source for their growth. Deletion of the T2SS components *gspD* and *gspE* significantly reduced LipA secretion in *A. baumanni* and the mutated strains were incapable to grow on the fatty acids as a sole carbon source (Johnson et al., 2016). The virulence function of T2SS in *A. baumanni* has been demonstrated in the *G. mellonella* and pulmonary infection models (Harding et al., 2016). The two secreted proteins LipA and CpaA, require specific chaperones for secretion and the respective genes are encoded adjacent to their effectors (Harding et al., 2016). A catheter-associated UTI (CAUTI) model demonstrated the uropathogenic potential of *A. baumannii* strain UPAB1 and highlighted the role of T2SS in virulence (Jackson-Litteken et al., 2022). A proteomic study unraveled the T2SS effector InvL that belongs to the intimin-invasin family and is required for *A. baumannii* uropathogenesis (Jackson-Litteken et al., 2022).

The type IV secretion system (T4SS) are involved in conjugative transfer of nucleic acids, in DNA uptake and release, and also in delivery of bacterial effector proteins into host (Morris et al., 2019; Sgro et al., 2019). However, the role of T4SS in host-pathogen interaction has yet to be explicated in *A. baumanni* (Smith et al., 2007; Liu et al., 2014). In gram-negative bacteria, type V (T5SS) plays a crucial role in biofilm formation, attachment to extracellular matrix components, and contributes to the survival of bacteria in systemic infection (Henderson et al., 2000; Bentancor et al., 2012a). In *Acinetobacter*, 5 subdivisions of T5SS family proteins have been classified (Bentancor et al., 2012a). The potential of T5SS autotransporter, Ata as a vaccine candidate was evaluated in a study, where antibodies against Ata were found to promote opsonophagocytic killing of *A. baumannii* by polymorphonuclear cells and also reduced attachment of *A. baumannii* cells to type iv collagen. In addition, passive administration of antisera to Ata, prior infection was found to significantly reduce the lung bacterial burden in a mice infection model (Bentancor et al., 2012b).

As an important nosocomial pathogen, many *A. baumanni* strains utilize type VI secretion system (T6SS) to deliver toxic effector proteins along with valine-glycine repeat protein G (VgrG) (forming part of the T6SS tip complex) towards neighbouring eukaryotic or bacterial cells. T6SS is generally involved in the ATP-dependent transport of effector proteins to mediate contact-dependent killing of competitor microbes (Mougous et al., 2006). The T6SS is composed of an envelope-spanning complex anchoring a cytoplasmic tubular edifice, and displays structural and functional similarity to bacteriophage tail (Shneider et al., 2013). The proline-alanine-alanine-arginine (PAAR), hemolysin co-regulated protein (Hcp), and VgrG necessary for T6SS function are delivered into target cells during their secretion process (Cianfanelli et al., 2016b). Bioinformatics analysis identified the T6SS in many *A. baumanni* strains. A functional T6SS was identified in *A. baumanni* strain M2 which showed the potent killing of competitor bacteria (Carruthers et al., 2013a). Later on, the strain M2 was classified as *A. nosocomialis* (Carruthers et al, 2013b; Harding et al., 2016). Diverse T6SS effectors group belonging to lipase (Tse1), DNase (Tse2), antibacterial effector (Tse3), and peptidoglycan hydrolase (Tse4) have been identified in *A. baumannii* ATCC 17978 (Weber et al., 2016; Ringel and Basler, 2017; Wijers et al., 2021). Ruiz et al. (2015) showed the functional T6SS in six pathogenic strains of *A. baumanni*, however, strain-specific virulence activity was observed (Repizo et al., 2015). In addition, the deletion of T1SS results in the suppression of the T6SS in cells grown in minimal medium, suggesting a possible cross-talk between these two systems (Harding et al., 2017b).

A fully functional T6SS genomic locus that mediates *E. coli* killing and favours host colonization during a *G. mellonella* model study was identified in strain DSM30011 (Repizo et al., 2015). Comparative genome analysis of many MDR strains of *A. baumanni* showed the truncated form of the T6SS locus, whereas, in some strains the T6SS genomic locus was completely absent (Wright et al., 2014; Jones et al., 2015). These evidences suggest more in-depth investigations of T6SS in diverse *A. baumannii* strains and also correlate its role in bacterial virulence. A large self transmissible resistance plasmid, equipped with T6SS regulators was found to supress the expression of the T6SS. Spontaneous loss of the plasmid resulted in the expression of the functional T6SS (Weber et al, 2015). Cells that become T6SS positive are, however, susceptible to killing under oxidative stress, which is mediated by host neutrophils. In a recent study, it was found that, *A. baumannii* employs a small RNA, AbsR28 to supress the T6SS that confers fitness under host-mediated oxidative stress (Bhowmik et al., 2022).

## Role of adherence and motility in virulence

Many pathogenic bacteria generally show distinct behavior of adherence and motility that are often intimately linked by complex regulatory networks. It is well known that the intrusion of a bacterium needs cell-to-cell adhesion for the establishment of infection (Falagas et al., 2007). The success of a pathogen often depends on its ability to adhere to biotic and abiotic surfaces. For example, attachment to epithelial cells will lead to the internalization of the bacteria into the host cell, and attachment to urinary catheters will decide whether the bacteria will be able to establish a chronic infection or not. The impressive ability of *A. baumannii* to attach to and survive on abiotic surfaces in hospital settings has played a significant role in the success of this bacterium as a nosocomial pathogen.

Several factors that mediate attachment to biotic and abiotic surfaces includes Bap (Biofilm Associated Proteins), Csu pili, and outer membrane proteins, such as OmpA, CarO, OmpW, and Omp33-36 etc. (Brossard and Campagnari, 2011; Rumbo et al., 2014; Labrador-Herrera et al., 2020; Gil-Marqués et al., 2022). In *A. baumanni*, Bap_ab_ play a crucial role in biofilm formation and maturation. In addition, they also help in adherence to host epithelial cells (Brossard and Campagnari, 2011). The protein is prevalent in *A. baumanni* strains including clinical and avirulents strains collected from different geographical regions (Liu et al., 2022). A study confirmed the involvement of Bap in modulation of cell surface hydrophobicity(CSH) which further support bacterial adherence to epithelial cells (Brossard and Campagnari, 2011). Additionally, the role of *blaPER-1* a broad range β-lactamase gene contributing to biofilm formation is not fully established and its distribution among all clinical isolates is under investigation (Lee et al., 2008).

Any mutation that renders these attachment factors non-functional, severely affect the cells adhesion/attachment ablity, which in turn affectits virulence. The direct relationship between motility and virulence has been established in several bacteria such as *E. coli, Salmonella* spp., and *P. aeruginosa* (Josenhans and Suerbaum, 2002). Similarly, in *A. baumannii*, a hypermotile isolate having a mutation in histone-like nucleoid structuring (H-NS) protein, was found to be more virulent than the parental strain (Eijkelkamp et al., 2013). Such studies indicates that motility play a significant role in the virulence of *A. baumannii*.

As mentioned before when discussing the role of pili, *A. baumanni* strains show pili-dependent twitching motility and surface-associated motility. In a *Galleria mellonella* infection model, surface-associated motility which is an appendage-independent form of movement, was found to play a more important role in virulence than twitching motility (Corral et al., 2021). Surface-associated motility is probably driven by the extrusion of extracellular polymeric substances and is associated with the synthesis of 1,3-diaminopropane (Skiebe et al., 2012), lipooligosaccharide production (McQueary et al, 2012), outer membrane and natural competence proteins (Blaschke et al., 2021), and proteins associated with superfamilies of efflux pumps (Pérez-Varela et al., 2019). The regulators of surface-associated motility include cyclic diguanylate (Ahmad et al., 2020), quorum sensing (Clemmer et al., 2011), CheW/Che Aanalogs (Corral et al., 2020), and EnvZ/OmpR -two component system (Tipton and Rather, 2017). *A. baumannii* strain AYE exhibits twitching motility, strain ATCC 17978 exhibits only surface-associated motility, whereas, the strain MAR002 shows both motility behaviours (Eijkelkamp et al., 2011; Skerniškyte et al., 2019).

In a study by Skiebe et al. (2012), they found that out of 83 A*. baumannii* clinical isolates, only one was found to be non-motile. In addition, in a screening of *A. baumannii* ATCC17978 transposon mutants they found two non-motile mutant that carry insertion mutation in *dat* and *ddc* gene, responsible for the synthesis of 1, 3-diaminopropane (DAP). Supplementing the media with DAP reverted the motility defect. This study thereby established the role of DAP in surface associated motility (Skiebe et al., 2012).

Various other factors like quorum sensing, iron, salinity, two-component system and lipopolysaccharides have been shown to affect the motility behaviour (Wood et al., 2018). Similarly, cell surface hydrophobicity enables the microorganism to attach to the surface hydrocarbons of cells, which further aids the survival of the organism (Krasowska and Sigler, 2014). Additionally, pathogens are able to control their surface hydrophobicity as per environmental conditions and also as per the requirement of growth phases (Khelissa et al., 2017a). The relation between CSH and pathogenicity has been studied in many pathogens, which shows that it has the ability to modulate virulence. A study with *A. baumanni* showed that CSH enhanced bacterial adherence to abiotic surfaces which further enhanced the biofilm formation. The increased biofilm formation in turn aids in the survival of *A. baumannii* in hospital setting (Pour et al., 2011). Another study illustrated that *A. baumanni* strain lacking a redox protein thioredoxin-A was more hydrophobic and readily taken up by macrophage (May et al., 2019), however, its confirmation by the *in vivo* model is still required.

## Regulation of virulence

A Tet-R family of transcription factors is involved in regulating phenotypic switching between opaque and translucent forms (Chin et al., 2018). These morphotypes were regulated by the alterations in ABUW_1645 expression, which control the switch gene expression, capsule biosynthesis, and also *in vivo* pathogenesis (Chin et al., 2018). The translucent form is generally avirulent, whereas, the opaque form exhibits resistance to antimicrobial peptides, ROS, lysozyme, and various disinfectants (Chin et al., 2018). Moreover, the translucent form is generally linked to biofilm formation, reduced capsule formation, and also upregulation of nutrient and catabolism-associated genes (Chin et al., 2018).

Disruption of a global regulator, H-NS in various clinical isolates increases colistin resistance, adherence to epithelial cells, and also increased *in vivo* virulence in *C. elegans* (Deveson Lucas et al., 2018). Disruption of H-NS genes also dysregulated various genes associated with OMP, T5SS, and biosynthesis of fatty acids (Eijkelkamp et al., 2013). Similarly, TCS also controls the phenotypic switching by modulating the transcriptional control, particularly membrane-bound sensor kinases and DNA-binding transcriptional regulator (Zschiedrich et al., 2016). In *A. baumanni*, a total of 20 TCSs have been identified each with distinct functionality (Liou et al., 2014). In *A. baumannii*, TCS are directly involved in the genetic expression in response to environmental signals and regulates the efflux pump, which is implicated in resistance to various antibiotics including aminoglycosides and tigecycline (Adams et al, 2018; De Silva and Kumar, 2019). These TCSs consist of sensor kinases embedded in cytoplasmic membranes that are stimulated by extracellular and interacellular stimuli (Groisman, 2016). External stimuli like osmotic pressure and pH initiate the phosphorylation event followed by a conformational change of transcription factor, contributing to DNA binding, and transcription (Kröger et al, 2016).

The TCS, AdeRS controls the RND efflux pumps like AdeABC, AdeFGH, and AdeIJK that showed their association with drug resistance against tetracyclines, aminoglycosides, tigecycline, erythromycin, and chloramphenicol (Giles et al., 2022). Another TCS, BaeSR, controls the expression of efflux pumps, whereas the BfmRS TCS regulate the morphology, attachment to biotic and abiotic surfaces, and biofilm expression (Lin and Lan, 2014). A newly discovered TCS, CheAY is associated with virulence and biofilm formation (Chen et al., 2017). The TCS PmrAB is involved in polymyxin B and colistin resistance through the gene modifications associated with lipopolysaccharides (Beceiro et al., 2011). In response to environmental stimuli, the TCS ompR-envZ regulates the porin expression and its mutation increased the switching frequency from opaque to translucent (Tipton and Rather, 2017). The TCS-component AbaI regulates the quorum sensing, PmrAB regulates the lipid-A metabolism, CheAY pili formation, and GacAS are responsible for the pathogenesis and immune evasion (Bhuiyan et al., 2016).

Among these, GacAS is critically involved in pathogenesis of bacteria, and the interactions between the host and pathogens. Disruption of *gacA* or *gacS* eliminates the ability to cause infection (Cerqueira et al., 2014). GacS interact with membrane sensor kinase especially at histidine and aspartic acid residue to induce the transcriptional regulator GacA phosphorylation (Cerqueira et al., 2014). Additionally, GacAS regulates many genes such as *ompA, csu* and *motB*, however, its *in vivo* regulation is still unclear. Among various transcription factors, H-NS are involved in the silencing of horizontally acquired genes/or AT-rich genes, thereby minimizing their harmful effects (Eijkelkamp et al., 2013).

Among TCS, BfmRS is required for transcriptional upregulation of capsule in response to antibiotics and is critical for various functions relevant to nosocomial disease (Geisinger and Isberg, 2015). The activation of BfmRS signaling augmented the virulence in *A*. *baumannii* and also regulates several stress-related pathways. Recently, the role of the BfmRS system in controlling siderophore biosynthesis and transport, and type IV pili production was documented (Palethorpe et al., 2022). BfmR binds to various stress-related promoter regions and directly activates the transcription of some stress-related genes. A reduction in biofilm formation (Tomaras et al., 2008), human ascites fluid and serum survival (Russo et al., 2016), reduced resistance to antibiotics (Russo et al., 2016), and fitness in the animal host (Wang et al., 2014) were observed in bacterial strains lacking BfmR.

The RNA chaperone Hfq also regulates bacterial virulence in many pathogens. Hfq mutation reduced the bacterial growth rate, changes in motility, membrane protein levels, and biofilm formation and also affects the *in vivo* virulence in *Salmonella* and *E. coli* (Bossi et al., 2008). In *Acinetobacter*, Hfq possesses an elongated C-terminal end, however, its functionality remains unknown (Kuo et al., 2017). In *A. baumanni, hfq* mutants display enhanced sensitivity to environmental stresses, reduced OMV, fimbriae and biofilm formation, and also it hampers the adhesion and invasion in eukaryotic cells (Kuo et al., 2017).

## Immune response

In response to *A. baumanni* infection, activation of neutrophils plays a key role to protect the host, especially in respiratory infections (Van Faassen et al, 2007). Upon infection, neutrophils are recruited at the site of infection, as early as 4 hours followed by a surge in infiltrating neutrophils, that reaches its peak after 24 hours post infection. The host factors that mediates neutrophilrecruitment are interleukin-1, tumor necrosis factor (TNF-α), keratinocyte chemoattractant protein (KC/CXCL1), macrophage inflammatory protein (MIP)-1, MIP-2/CXCL2, and monocyte chemoattractant protein. In neutrophil depleted host, the severity of infection and lethality was higher, and was associated with a delayed production of the neutrophil recruitment and activation factors (Henk van et al., 2007; García-Patiño et al., 2017). The protective mechanism of neutrophils involves TLR-mediated phagocytosis, complement-mediated receptor binding, and a neutrophil extracellular traps (NETs) formation (Nordenfelt and Tapper, 2011). After phagocytosis, bacterial killing depends on ROS and granular fusion, which leads to the release of various antimicrobial molecules, human defensins, and lysosomes into the phagosome (Qiu et al., 2009; Harding et al., 2017a). Kamoshida et al. (2016) showed that *A. baumanni* promotes their dissemination in an IL-8-dependent manner by adhering to the neutrophil’s surfaces.

The formation of NET involves antimicrobial proteins and peptides, neutrophil elastase, myeloperoxidase, IL-37, and a mesh of chromatins, which has been associated to inhibit bacterial infection (Konstantinidis et al., 2016). However, detailed experimental evidence is still required to elucidate the induction of NET formation in response to *A. baumanni* infection (Kamoshida et al., 2015; Kamoshida et al., 2018). Among cytokines, IL-8 and TNF-α can stimulate neutrophils recruitment, however, TNF-α-mediated stimulation is generally concentration-dependent such as the release of cytokine and activation of MAP kinase (Kikuchi-Ueda et al., 2017). Other host factors (e.g., serum amyloid A/P and neutrophil phosphatase) also regulate cytokine secretion and neutrophil migration (Sun et al., 2014).

Apart from neutrophils, macrophages have a defensive function against bacterial infections and also promote neutrophil recruitment at the infection site (Qiu et al., 2012). Alveolar macrophages stimulate the level of IL-6, IL-10, TNF-α, and macrophage inflammatory protein-2 to protect against *A. baumanni* lung infection (Qiu et al., 2012). Macrophage depletion leads to an increase in bacterial burden and also reduced PIC secretion, however, the killing activity of macrophages is slower than that of neutrophils (Lázaro-Díez et al, 2017). Besides, natural killer (NK) cells are also a well-known defender against intracellular and extracellular bacteria, and viral infections (Paidipally et al., 2018). The NK cells-mediated bacterial clearance involves the release of neutrophil chemoattractant as well as keratinocyte productions, however, their role against *A. baumanni* infection is still unclear (Waggoner et al., 2016). Similarly, dendritic cells (DCs) link both innate and adaptive immune systems by acting as antigen-presenting cells (APC). The OMP ‘OmpA’ of *A. baumanni* activates the DCs to enhance the MAP kinase and NF-kB signalling to induce the CD4^+^ Th_1_ T-cell, and early apoptosis (Lee et al., 2007). However, *A. baumanni* may modulate the T-cell response through consistent production of ROS and it may lead to DCs death *via* mitochondrial targeting (Lee et al., 2010). In response to *A. baumanni* invasion, mast cells secrete the IL-8 and TNF-α, enhancing the neutrophil recruitment to the site (Kikuchi-Ueda et al., 2017).

Similarly, in response to *A. baumanni*, TLR signalling play an essential role in the host for recognition of pathogens by detecting porin proteins, peptidoglycan, lipoproteins, lipoteichoic acid, and CpG DNA motif (Noto et al, 2015). Activation of TLR2 and TLR4 in the presence of GPI-linked glycoproteins enhances the NF-kB signaling to release the proinflammatory cytokines including IL-1, IL-6, IL-8, IL-10, TNF-α, and IFN-γ (Moffatt et al., 2013). TLR2 knockout mice (TLR2 -/-) showed an increase in bacterial burden in the first 24 h of infection, whereas, Knapp et al., 2006 reported a lower bacterial load simultaneously (Knapp et al., 2006; Kim et al., 2014). These differences vary among *A. baumanni* isolates, however, a detailed investigation is still required. Interestingly, TLR4 knockout reduces the lethal effect of *A. baumanni* septicaemia by minimizing the septic shock caused by LOS which clearly illustrates that level of LOS and TLR4 signaling is linked to *A. baumanni* virulence (Lin et al., 2012).

*A. baumanni* isolates with phosphoethanolamine-modified lipid-A trigger the TLR4 signalling compared with unmodified LOS (Lin et al., 2012). On the other hand, LpxC inhibitor enhances the TLR2 signalling and reduces the NF-kB signalling and TNF-α secretion, promoting opsonophagocytic killing, thereby protecting against *A. baumanni*-lethal effects (Lin et al., 2012). TLR9 is an endolysosome receptor and is responsible for bacterial and viral DNA detection (Harding et al., 2017a). TLR9 stimulation enhances the activation NF-kB and release of PIC, whereas, TLR9-/- mice showed reduced secretion following *A. baumanni* infections (Noto et al., 2015). In response to *A. baumanni* infections, NLRP3 caspase-1/11 activation enhances the secretion of IL-1β and IL-18 from infected lung cells, thereby promoting tissue damage (Dikshit et al., 2017). The cytokine IL-17 has emerged as an interesting candidate for promoting granulopoiesis and inducing cytokine, chemokine, and antimicrobial peptide expression including GM-CSF and IL-8 (García-Patiño et al, 2017). Besides TLR signalling, NOD-like receptors including Nod1 and Nod2 are involved in PAMP recognition, and induce NF-kB signalling, however, their role in MAP-kinase activation is not clear (Bist et al., 2014).

Similarly, complement-mediated killing involves multiple soluble factors that promote the lysis of bacterial cells or other activities like opsonophagocytosis in a cascade manner. These pathways regulate the deposition of various complement factors on the bacterial cell surface, however, alternate complement pathways are also involved in *A. baumanni* killing in human serum (Kim et al., 2009; Bruhn et al., 2015). Few MDR-resistant strains of *A. baumanni* showed a certain level of resistance against these complement factors and therefore cause disease more severely (Bruhn et al., 2015). In human serum, an alternate complement pathway deposits factor C3 on the serum-sensitive bacterial strains, and these depositions are regulated by factor H which is a soluble component for recognizing host cell markers (Ferreira et al., 2010). In a study, Kim et al. (2009) showed that serum resistance in *A. baumannii* is mediated by acquisition of factor H on bacterial surface, as it interacts withouter membrane proteins, including OmpA. In another study, Koenigs et al. (2016) showed that *A. baumanni* plasminogen protein CipA promotes the C3b cleavage and degradation of fibrin networks *via* inhibiting the alternate complement pathway, however, detailed investigations are still required. Besides plasminogen proteins, genes contributing to *A. baumanni* cell envelope homeostasis also promote serum resistance in many clinical isolates.

Disruption of the gene encoding the pilus and biosynthesis regulator, BfmS (of the BfmRS TCS) enhance serum resistance (Geisinger et al., 2018). Similarly, capsule biosynthesis genes like *ptk, epsA*, and *mltB* contribute to serum resistance, indicating the role of the capsule in resisting the complement pathways mediated killing (Lees-Miller et al, 2013; Crépin et al., 2018). Despite several studies that have elucidated the role of different bacterial components in inducing the adaptive immune response, the role of Th1, Th2 or Th17 has yet to be elucidated (Pulido et al., 2018; Song et al., 2018). In some studies, it was observed that induced levels of antibody IgM, IgG, and cytokine-like IL-4, IL-17 enhance bacterial clearance and enhance the survival of the host (Luo et al., 2012; García-Quintanilla et al, 2014). Some *A. baumannii* clinical isolates can establish a protective intracellular niche for multiplication for a prolonged time. This protective intracellular compartment protects *A. baumannii* from normal degradative pathways and host immune responses, and also hinders antibiotic accessibility. This further enhances the *A. baumannii* persistence and mortality in susceptible patients (Rubio et al., 2022). Further extensive studies are required to elucidate the effect of different adaptive immune components on the clearance of *A. baumanni* infection.

## Conclusion

A number of *in vitro* and *in vivo* studies involving animal models have produced important information regarding the *A. baumanni* pathogenesis. Studies on the various acquisition systems like metal, nutrient and protein secretion systems have broadened the understanding of *A. baumanni* virulence. However, more extensive studies are still required on diverse secretion systems in *A. baumanni* to identify the genes linked to pathogenesis. Experimental approaches like whole genome sequening, transposon screening, and Tn-sequencing will provide a deep understanding of pathogenicity and might be useful for the development of novel antibiotics. In addition, due to the considerable difference between the genome of recent clinical isolates and the type strains, the use of the recent isolates will enable us with a contemporary understanding of the virulence and pathogenesis factors, important for the success of this pathogen. Antibiotic resistance in clinical isolates of *A. baumanni* is on the rise and the ability of the isolates to acquire resistance to various antimicrobial compounds demands to look for new methods for the discovery of novel drug targets and strategies, which may certainly avoid infection transmission and establishment. Expanding more studies on the comparative genomics of drug-resistant strains will facilitate a better understanding of novel transposons and mobilizable plasmids responsible for antibiotic resistance. Furthermore, analysis of phylogenetic diversity among clinical and nonclinical strains will be helpful to gain a comprehensive understanding of processes causing the evolution of this species as a global pathogen.

## Author contributions

Conceptualization: RS. Original draft writing: AS, AP, RS. Editing: RP, RS and YM. All authors contributed to the article and approved the submitted version.
